# Neuromuscular Mechanisms and Oxidative Stress in Skeletal Muscle Atrophy: Emerging Stem Cell and Gene-Based Therapeutic Strategies

**DOI:** 10.3390/muscles5010013

**Published:** 2026-02-10

**Authors:** Sathish Kumar Gunasekaran, Mandam Amzad Khan, Mehwish Mirza, Santhosh Shanthi Bhupathi, Mohamed Sheik Tharik Abdul Azeeze

**Affiliations:** 1 Department of Pharmacology, JSS College of Pharmacy, Mysore 570015, Karnataka, India; sathishg0207@gmail.com; 2 Department of Pharmacy Practice, Balaji College of Pharmacy, Anantapuramu 515001, Andhra Pradesh, India; mamzadkhan43@gmail.com; 3 Department of Biological Sciences, Seton Hall University, South Orange, NJ 07079, USA; mehwish.mirza@student.shu.edu; 4 WVU Cancer Institute, West Virginia University, Morgantown, WV 26506, USA; santhosh.nov8@gmail.com; 5 Institute of NeuroImmune Pharmacology, Seton Hall University, South Orange, NJ 07079, USA

**Keywords:** skeletal muscle atrophy, neuromuscular junction, mitochondrial dysfunction, ubiquitin-proteasome system, FOXO, NF-κB, stem cell therapy, gene therapy

## Abstract

Skeletal muscle atrophy emerges from intertwined neuromuscular and metabolic failures, in which neuromuscular junction destabilization, excitation contraction coupling defects, and mitochondrial dysfunction collectively intensify calcium dysregulation and drive the accumulation of reactive oxygen and nitrogen species (RONS), reinforcing proteolytic and catabolic signaling programs. To integrate recent evidence on the neuromuscular redox interface and highlight therapeutic strategies that target these interdependent drivers of atrophy. RONS-mediated activation of NF-κB and FOXO pathways accelerates ubiquitin proteasome and autophagy lysosome degradation, leading to motor unit loss. Stem cell therapies (satellite cells, MSCs, and iPSC progenitors) seek to restore regenerative potential but face hurdles in engraftment and reinnervation. Gene-based interventions, including antioxidant gene delivery, Nrf2 activation, RNA modulators, and CRISPR editing, offer new avenues but remain limited by safety and delivery barriers. Bioengineering platforms such as hydrogels, decellularized scaffolds, and extracellular vesicles provide architectural, trophic, and immunomodulatory support. Translational progress requires rigorous safety pipelines, mechanistic biomarkers of motor unit recovery, and modular combination regimens that integrate cells, genes, scaffolds, and rehabilitative input. By aligning neuromuscular biology with redox control, emerging strategies hold promise to rebuild innervated, fatigue-resistant muscle across acquired and genetic atrophy syndromes.

## 1. Introduction

Skeletal muscle atrophy is not just a loss of mass; it is a systems-level failure that couples neuromuscular disconnection with redox imbalance and catabolic reprogramming. Across conditions as diverse as disuse, aging (sarcopenia), denervation, cachexia, and genetic myopathies, patients experience weakness, fatigability, and impaired recovery after injury. Population-level data underscore its reach: contemporary meta-analyses estimate sarcopenia prevalence near 10% in community-dwelling older adults, rising sharply with frailty and multimorbidity [1]. Preventing or reversing muscle atrophy has become essential for maintaining independence in aging populations and improving outcomes in chronic disease. Across human and animal studies, the most consistent upstream driver of atrophy is neuromuscular dysfunction, characterized by early Neuromuscular junction (NMJ) fragmentation, denervation, reduced motor unit firing, impaired axonal transport, and synaptic instability triggered by inactivity or disease [2,3,4]. RONS are not merely bystanders in this process; they actively remodel synapse structure and transmission. Emerging work positions RONS as bidirectional regulators modulating synaptic proteins and transmitter release at physiological levels, while driving degeneration when redox balance is lost [5]. With age and inactivity, deficits in mitochondrial function within both muscle fibers and motor neurons converge with NMJ changes, creating a self-reinforcing loop of impaired excitation, reduced use, and progressive atrophy [6]. At the fiber level, excitation–contraction (EC) coupling becomes unreliable. Oxidative post-translational modifications of the ryanodine receptor (RyR1) destabilize its closed state, promoting sarcoplasmic Ca^2+^ leak, energetic inefficiency, and further oxidant production by mitochondria [7,8]. These Ca^2+^ and redox disturbances blunt force generation and activate proteolysis, knitting the mechanical and metabolic phenotypes of atrophy into a single pathophysiological fabric. Oxidative stress, in turn, is multifactorial in origin. Mitochondrial electron transport chain slippage, NADPH oxidases (NOX2/NOX4), uncoupled nitric oxide synthase, and inflammatory oxidant sources elevate RONS during disuse, denervation, and catabolic illness [9,10,11]. Sustained oxidative stress damages lipids, proteins, and DNA, impairs mitochondrial biogenesis, and disrupts organelle cross-talk effects that magnify when innervation is compromised and contractile activity is low [12]. Mechanistically, redox cues feed forward into the transcriptional control of atrophy: the ubiquitin proteasome system (UPS) is induced; autophagy lysosome flux is reprogrammed; and central transcriptional nodes, including FOXO and NF-κB, tilt the cell toward net protein loss [13,14,15]. In short, neuromuscular disconnection and oxidative stress are not parallel stories; they are the same narrative told from different angles. The clinical corollary is straightforward. Exercise and reloading can stabilize NMJs, restore redox signaling, and partially rescue function, but they are not always feasible or sufficient in neurodegenerative disease, severe disuse, or advanced sarcopenia [4,16]. Hence, the turn toward biological therapies that can rebuild or reprogram tissue. Two frontiers now dominate the conversation: stem cell-based regeneration and gene-based modulation. On the cellular front, satellite cells (muscle stem cells, MuSCs) are indispensable for regeneration, yet their pool and functionality erode with age and chronic disease through niche disruption, metabolic drift, and heightened inflammatory signaling [17]. Contemporary reviews synthesize how altered cross-talk between MuSCs and their microenvironment, endothelium, fibro-adipogenic progenitors, immune cells, and motor axons drives impaired repair and atrophy [17]. Mesenchymal stromal/stem cells (MSCs) and induced pluripotent stem cells (iPSCs) broaden the toolkit: MSCs offer paracrine immunomodulation and pro-regenerative secretomes, while iPSCs enable scalable myogenic progenitors and patient-specific disease modeling, with recent proof-of-concept for generating transplant-competent muscle stem cells that can contribute to the MuSC reservoir in vivo [13,18,19]. Still, the field grapples with homing, survival, myofiber fusion, long-term engraftment, and the need to reestablish innervation, not just myogenesis [6,20]. Gene-based strategies are moving in parallel. Antioxidant gene delivery, catalase or SOD variants targeted to mitochondria, Nrf2 pathway activation, and engineered decoys or repressors for pro-atrophy transcriptional programs aim to reset redox and catabolic tone in muscle fibers [21,22]. Inherited myopathies have catalyzed clinical momentum for systemic adeno-associated virus (AAV) delivery to skeletal muscle. The most visible milestone is delandistrogene moxeparvovec (Elevidys), an AAVrh74 micro-dystrophin gene therapy that received initial U.S. approval in 2023 and expanded indications in 2024, evidence that large-muscle gene transfer can achieve regulatory-grade efficacy signals, while also exposing real safety and durability challenges [23]. CRISPR-based editing and base-editing approaches for dystrophin restoration and other myofiber targets have advanced to preclinical-to-early clinical stages, raising the prospect of disease-modifying interventions that may also secondarily stabilize NMJs by restoring fiber integrity [24,25]. A critical insight emerging across these domains is synergy. Combining stem cells with gene modulation, bioengineered scaffolds that provide aligned architecture and pro-innervation cues, and pharmacological support to control inflammation and oxidative stress appears necessary to rebuild a functional motor unit, not just bulk tissue. Recent tissue-engineering advances, such as decellularized matrices, hydrogel fibers, and anisotropic scaffolds, improve MuSC retention and orientation, enhance vascularization, and may facilitate reinnervation when paired with activity-based rehabilitation [26,27,28]. For translational success, these constructs must integrate electrical and mechanical signaling, restore mitochondrial fitness, and maintain redox homeostasis during the vulnerable engraftment window [6].

This review brings these threads together. We first dissect the neuromuscular mechanisms of atrophic motor neuron pathology, NMJ instability, and EC coupling defects, then map how oxidative stress is generated and propagated within this network. We next analyze molecular pathways that link neuromuscular dysfunction to catabolism (UPS, NF-κB, FOXO, and Ca^2+^ homeostasis), before evaluating stem cell and gene-based therapeutic strategies, their points of convergence, and the bioengineering frameworks that may make them work in patients. Along the way, we stay close to recent evidence and keep a clear line of sight on translation: preclinical models, safety, manufacturing, and the current clinical pipeline. The end goal is simple: a mechanistic playbook that aligns neuromuscular biology with redox control to design therapies that rebuild innervated, fatigue-resistant muscle.

## 2. Neuromuscular Mechanisms in Skeletal Muscle Atrophy

### 2.1. Motor Neuron Degeneration: A Primary Driver in Many Atrophic Syndromes

Motor neurons are more than signal wires; they provide trophic support that maintains muscle phenotype. When motor neurons degenerate in ALS, spinal muscular atrophy (SMA), aging-associated motor unit loss, or focal nerve injury, target muscle fibers rapidly atrophy [29]. Progressive motor unit loss reduces neural input, decreases contractile activity, and initiates a cascade of proteolytic and metabolic changes in muscle that favor net protein loss. Elevidys (delandistrogene moxeparvovec) has been evaluated in multiple AAV micro-dystrophin trials with randomized, placebo-controlled designs in ambulant boys with Duchenne muscular dystrophy, including dose-escalation and age-stratified cohorts. In the EMBARK study, the intended dose approximately 1.33 × 10^14^ vg/kg increased micro-dystrophin expression to roughly 40% of normal on average, with a modest age-dependent NSAA benefit in 4–5-year-old participants but not in older boys [30]. Additionally, across trials, serious treatment-emergent toxicity has mainly involved acute liver injury and immune-mediated myositis, which are usually medically manageable with corticosteroids, though rare fatal liver failure has been reported in broader post-marketing experience. Longer-term durability remains uncertain: follow-up beyond 2–3 years is limited, and registry-based programs such as EXPEDITION are still accruing data on sustained motor function and late adverse events [31]. The experimental literature documents that denervation alone is sufficient to trigger robust atrophy through transcriptional induction of ubiquitin ligases and metabolic reprogramming [32,33,34]. Mechanisms of motor neuron vulnerability are multifactorial. In neurodegenerative diseases (ALS, SMA), intrinsic cell-autonomous processes, such as protein aggregation, impaired RNA metabolism, and defective axonal transport, combine with non-cell-autonomous stressors, such as glial dysfunction, neuroinflammation, and impaired neurotrophic support. Motor neurons provide continuous trophic support that preserves muscle fiber phenotype; experimental denervation in rodents is sufficient to induce rapid atrophy with up-regulation of ubiquitin ligases (atrogin-1, MuRF1) and metabolic reprogramming toward catabolism. In ALS and SMA models, axonal transport defects, mitochondrial dysfunction in distal terminals, and impaired neurotrophic signaling cooperate to accelerate synaptic withdrawal and fiber loss [35]. Age-related motor unit loss shows similar features, with early mitochondrial dysfunction at neuromuscular terminals and reduced activity-dependent antioxidant responses preceding overt denervation. Thus, preserving motor neuron health and axonal transport is a necessary complement to muscle-targeted therapies; promoting myogenesis alone risks generating non-innervated fibers that remain functionally weak. These mechanisms reduce axonal transport of mitochondria and synaptic cargoes, impair terminal energy supply, and predispose the motor terminal to synaptic failure and withdrawal. Even in age-related motor unit loss, emerging work points to mitochondrial dysfunction within axon terminals and synaptic mitochondria as early, upstream pathology [32,36]. Preserving motor neuron health (axon transport, mitochondrial fitness, neurotropic signaling) is a necessary complement to muscle-targeted approaches. Interventions that simply augment myogenesis without restoring innervation risk forming non-innervated fibers that remain functionally compromised [33].

### 2.2. Neuromuscular Junction Instability: Structural and Molecular Failure

The NMJ is the functional synapse between the motor axon and the muscle fiber. NMJ instability is a consistent, early feature in disuse, aging (sarcopenia), denervation models, and several neuromuscular diseases. Morphologically, instability manifests as fragmentation of post-synaptic acetylcholine receptor (AChR) clusters, thinning or retraction of presynaptic terminals, and loss of alignment between pre- and post-synaptic specializations. Functionally, this produces a reduced safety factor for transmission, intermittent failure at high firing rates, and progressive denervation of motor units. Systematic reviews across human and animal studies highlight NMJ transmission failure as a key contributor to age-related weakness [2,6,37]. Molecular drivers of NMJ destabilization include loss or dysfunction of agrinLRP4MuSK signaling (which organizes postsynaptic AChR clustering), altered synaptic extracellular matrix, inflammatory mediators, and redox-dependent modification of synaptic proteins. Inactivity and denervation decrease nerve-derived trophic factors, impair Schwann cell support, and alter the local immune milieu, all of which accelerate synapse dismantling [38]. Recent human bed-rest and disuse studies show rapid NMJ morphological alterations within days to weeks of inactivity, underscoring the dynamic of the synapse [3,39]. Importantly, the NMJ is a redox-sensitive structure. RONS at synaptic sites modulate presynaptic transmitter release and postsynaptic receptor function at physiological levels, but chronic or excessive RONS cause protein oxidation, impair agrin signaling, destabilize AChR clusters, and promote terminal retraction. This positions oxidative stress not as a parallel phenomenon but as a direct molecular effector of synaptic loss [37,40]. Therapeutic implication: interventions that preserve or restore agrin MuSK signaling, stabilize extracellular matrix components, reduce synaptic oxidative damage, or promote terminal reinnervation through neurotrophic support, electrical stimulation, or engineered scaffolds are promising ways to maintain functional motor units during aging and disease [39,41,42,43].

### 2.3. Altered EC Coupling and Calcium Dysregulation

EC coupling links membrane depolarization to Ca^2+^ release from the sarcoplasmic reticulum (SR) via physical interaction of the voltage-sensing dihydropyridine receptor (Cav1.1) and the type 1 ryanodine receptor (RyR1). Loss of precise control of SR Ca^2+^ release both reduces contractile force and activates catabolic signaling cascades [44,45]. Two interconnected pathological processes stand out: RyR1 post-translational modification and SR Ca^2+^ leak. Oxidative and nitrosative modifications (S-nitrosylation, carbonylation) and abnormal phosphorylation of RyR1 increase its open probability and promote pathological SR Ca^2+^ leak. Chronic leak raises resting cytosolic Ca^2+^, activates Ca^2+^-sensitive proteases and phosphatases, and burdens mitochondria with Ca^2+^ uptake, precipitating bioenergetic failure and further ROS production. Recent studies in aging and denervation link RyR1 oxidation and calstabin (FKBP12) dissociation to impaired tetanic Ca^2+^ release and reduced specific force [8,46]. Disrupted transverse (T)-tubule integrity and coupling proteins. Structural disarray of the T-tubuleSR junctions, altered expression of associated scaffolding proteins, or pathological remodeling after denervation reduce the efficiency of depolarization-triggered Ca^2+^ release. This manifests as reduced twitch and tetanic force and heightened fatigability even in fibers that retain contractile proteins [47,48]. The downstream consequences of chronic Ca^2+^ dysregulation are twofold: (a) activation of proteolytic systems (calpains, caspases) and transcriptional regulators that promote muscle protein breakdown; (b) mitochondrial Ca^2+^ overload leading to permeability transition pore (PTP) opening, loss of membrane potential, and amplified ROS release. Denervation studies explicitly link heightened Ca^2+^ sensitivity of the PTP and mitophagy activation to atrophy [34,49]. Therapeutic implication: stabilizing RyR1 gating (pharmacologically or by preventing oxidative modification), preserving T-tubule integrity, and buffering pathological cytosolic Ca^2+^ during denervation or disuse are attractive strategies to interrupt the feed-forward loop between Ca^2+^ dysregulation and oxidative damage [8,46].

### 2.4. Cross-Talk: How Motor Neuron Loss, NMJ Failure, and EC Coupling Feed Each Other

**Figure 1:**
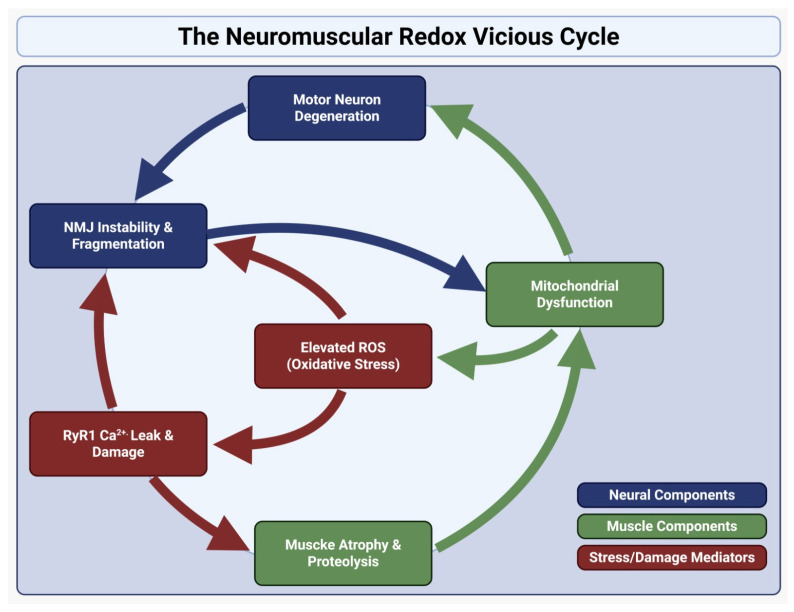
Neuromuscular redox vicious cycle. Schematic representation of the feed-forward cycle in skeletal muscle atrophy. Motor neuron degeneration, NMJ instability, and RyR1-mediated Ca^2+^ leak converge on mitochondrial dysfunction to amplify oxidative stress, accelerating synaptic dismantling and proteolysis. Retrograde injury further destabilizes NMJ input, while oxidative modification of RyR1 drives Ca^2+^ overload and mitochondrial failure, reinforcing a self-perpetuating cycle of redox stress, fiber degeneration, and muscle atrophy.

These three nodes are not independent. Figure 1 and Table 1 represent how motor neuron dysfunction reduces patterned electrical activity and neurotrophic input, initiating NMJ dismantling and myofiber inactivity [50]. Reduced contractile activity impairs mitochondrial turnover and antioxidant responses, increasing intracellular RONS that modify RyR1 and other EC coupling proteins. RyR1 leak and mitochondrial dysfunction produce metabolic insufficiency and local oxidative microenvironments that promote further synapse instability and retrograde signaling to motor neurons [51]. In summary, loss of neural input initiates synaptic withdrawal and myofiber inactivity, which in turn promotes mitochondrial dysfunction and pathological Ca^2+^ handling. These alterations amplify oxidative stress, further destabilizing neuromuscular junctions and exacerbating motor neuron injury. This feed-forward cycle contributes to the rapid progression from denervation to irreversible skeletal muscle atrophy across multiple pathological contexts. It is well showcased in Table 1 [8,34,37].

### 2.5. Clinical and Experimental Evidence That Ties Mechanisms to Phenotype

Denervation models: Peripheral nerve transection or chronic disuse models show rapid induction of atrophy genes (atrogin-1, MuRF1), mitochondrial ROS increases, RyR1 dysfunction, and NMJ morphological breakdown. These models demonstrate causality and identify therapeutic window timing [34,61].

Aging and sarcopenia: Human and rodent studies show motor unit loss, NMJ fragmentation, reduced motor unit firing rates, and RyR1 post-translational modifications with age; interventions that restore activity patterns or redox balance partially recover function [3,6,37].

Neurodegenerative disease: SMA and ALS provide paradigms where primary neuron loss precipitates severe atrophy; conversely, muscle-targeted therapies that improve fiber integrity can secondarily stabilize NMJs. Recent SMA reviews emphasize the need for combined neuron- and muscle-directed strategies [33,36].

## 3. Role of Oxidative Stress in Muscle Degeneration

Oxidative stress in skeletal muscle atrophy is best understood as an imbalance between the production of RONS and the cell’s capacity to detoxify them. Under physiological conditions, RONS serve signaling roles in exercise adaptation and synaptic plasticity, but when production overwhelms antioxidant defenses, oxidative modifications to lipids, proteins, and nucleic acids accumulate and cellular homeostasis collapses. A number of contemporary reviews and experimental studies since 2022 emphasize this duality and show that chronic or excessive RONS are central drivers of disuse, denervation, disease, and age-associated muscle loss [62].

### 3.1. Sources of Reactive Oxygen and Nitrogen Species in Muscle

The primary intracellular sources of RONS in skeletal muscle are multiple and context-dependent. Mitochondria are a major origin: electron leak from complexes I and III of the electron transport chain (ETC) produces superoxide that is rapidly converted to hydrogen peroxide, particularly when respiration is impaired, or proton motive force is altered. Several recent reviews and mechanistic studies highlight how inactivity, denervation, and aging increase mitochondrial electron leak and reduce the efficiency of oxidative phosphorylation, thereby elevating basal ROS emission [10,11]. In addition to mitochondria, nicotinamide adenine dinucleotide phosphate (NADPH) oxidases, principally NOX2 and NOX4, in muscle generate regulated bursts of ROS that modulate contraction and signaling but, when dysregulated, contribute to pathological oxidative load. The complete list of sources are provided in Table 2. Several studies clarify isoform-specific roles of NOX enzymes in skeletal muscle physiology and in disease states such as insulin resistance and denervation-induced atrophy [9,63]. Finally, uncoupled nitric oxide synthases (nNOS/eNOS) and inflammatory enzyme systems can produce secondary RONS (peroxynitrite, nitrogen dioxide) that nitrosylate proteins and disrupt redox-sensitive signaling nodes, and the literature has increasingly emphasized altered NO homeostasis as a contributor to sarcopenia and metabolic-disease-related muscle loss [64,65].

### 3.2. Oxidative Damage to Proteins, Lipids, and DNA

Once generated, RONS inflict diverse molecular damage. Lipid peroxidation alters membrane fluidity and the function of membrane-bound proteins, including those at the NMJ and T-tubule system. Protein oxidation and carbonylation degrade contractile and metabolic enzymes, and promote misfolding and aggregation that burden proteostasis pathways. Oxidative lesions in nuclear and mitochondrial DNA impair transcriptional programs and mitochondrial protein synthesis, further eroding bioenergetic capacity. Recent experimental work and targeted reviews catalog these changes in denervation and disuse models, showing early rises in protein carbonyls, 4-hydroxynonenal adducts, and oxidative mtDNA damage that temporally precede maximal fiber loss [10,62].

### 3.3. Mitochondrial Dysfunction and Redox Imbalance

Mitochondrial dysfunction acts as both a source and a consequence of oxidative stress in atrophying muscle, forming a self-amplifying loop. Disrupted fusion–fission dynamics and impaired mitophagy allow damaged mitochondria to accumulate, increasing ROS production and diminishing ATP output. In parallel, Ca^2+^ overload and oxidative injury to inner-membrane proteins promote permeability transition pore opening, leading to membrane depolarization and ROS bursts. Evidence shows that mitophagy (PINK1/Parkin and receptor-mediated pathways), mitochondrial biogenesis (PGC-1α), and Nrf2-driven antioxidant defenses are all compromised during disuse and aging, creating conditions that accelerate bioenergetic decline [66,67]. Functionally, mitochondrial defects reduce fatigue resistance and impair local ATP availability needed for synaptic vesicle cycling and sarcoplasmic reticulum (SR) Ca^2+^ re-sequestration, linking organelle failure to both contractile and synaptic pathology.

### 3.4. Cross-Talk Between Oxidative Stress and Neuromuscular Impairment

Redox imbalance also reshapes intracellular signaling to favor proteolysis. Oxidative cues activate NF-κB, drive FOXO nuclear entry, and enhance the sensitivity of the ubiquitin–proteasome and autophagy pathways to catabolic induction. Recent studies show that RONS upregulate key E3 ubiquitin ligases (atrogin-1/MAFbx and MuRF1) in denervation and disuse, and that boosting antioxidant capacity blunts this transcriptional atrophy program [62,68]. The neuromuscular compartment motor axons, terminals, Schwann cells, and postsynaptic structures is highly vulnerable to redox disturbances. Oxidative modifications of synaptic proteins, extracellular matrix components, and agrin MuSK signaling destabilize acetylcholine receptor clustering and lower the safety margin for transmission. Experimental work shows that mitochondrial dysfunction in either neurons or muscle generates ROS microdomains that impair presynaptic release and promote terminal withdrawal, whereas enhancing antioxidant defenses preserves NMJ structure and delays denervation. Thus, oxidative stress drives not only fiber atrophy but also the progressive loss of innervation that accelerates muscle wasting [69,70,71,72,73,74]. Redox modifications of RyR1 and its stabilizing partners compromise excitation–contraction coupling by increasing SR Ca^2+^ leak and sustaining cytosolic Ca^2+^ overload. This dysregulation activates calpains, calcineurin, and other Ca^2+^-dependent proteases, while burdening mitochondria through excessive Ca^2+^ uptake. Recent studies highlight RyR1 S-nitrosylation/carbonylation and loss of stabilizing subunits as key defects in aging and disuse, and show that preventing RyR1 oxidation lowers Ca^2+^ leak and suppresses downstream proteolytic signaling [66,75]. Antioxidant pathways and redox sensors offer tractable therapeutic targets. Nrf2 orchestrates glutathione synthesis, superoxide detoxification, and other cytoprotective programs, positioning it as a central regulator of muscle redox balance and mitochondrial quality control. Recent preclinical studies show that pharmacological Nrf2 activation and enhancement of mitochondrial antioxidant capacity preserve NMJ integrity and mitigate denervation-induced atrophy, providing proof of concept that targeted redox modulation can modify disease progression [67]. Oxidative stress in skeletal muscle atrophy arises from multiple sources and is mechanistically central to both fiber degeneration and neuromuscular disconnection. Mitochondrial dysfunction, NOX activity, and dysregulated NOS generate persistent RONS that damage macromolecules, impair proteostasis, and destabilize synapses and EC coupling. Therapeutic strategies that restore mitochondrial quality control, modulate NOX/NOS signaling, and activate Nrf2-driven antioxidant defenses coordinated with interventions that preserve innervation represent a plausible path to slowing or reversing atrophy. Recent studies provide strong mechanistic support for such targeted redox-based approaches and highlight candidate pathways for translation [10,64,66].

## 4. Molecular Pathways Linking Neuromuscular Dysfunction and Oxidative Stress

### 4.1. Ubiquitin–Proteasome and Autophagy–Lysosome Systems

UPS is the major driver of ubiquitin-dependent proteolysis in atrophying muscle and a central integrator of neuromuscular and redox signals that trigger myofibrillar loss. Denervation, disuse, inflammation, and cachexia all converge on upregulation of the muscle-specific E3 ligases atrogin-1/MAFbx and MuRF1, increased ubiquitination of structural proteins, and heightened 26S proteasome activity. Recent work shows that UPS activation reflects both reduced PI3K–Akt signaling, which releases FOXO-mediated transcription of E3 ligases, and redox-sensitive kinase pathways that further amplify this program. Pharmacologic or genetic inhibition of UPS components attenuates atrophy in animal models, underscoring its causal role and supporting targeted modulation of E3 ligases and proteasome regulation as a viable therapeutic strategy [13,76]. These molecular pathways operate in interconnected feedback loops that accelerate neuromuscular decline. Loss of patterned neural input suppresses IGF-1/Akt signaling and mitochondrial biogenesis, facilitating FOXO-driven induction of E3 ligases. In parallel, reduced contractile activity and nerve terminal mitochondrial dysfunction generate ROS microdomains that activate NF-κB, oxidatively modify RyR1 and other EC coupling proteins, and heighten sensitivity of the UPS and autophagy machinery to catabolic cues. Both proteolytic systems are intrinsically redox-responsive, as oxidative damage increases misfolded protein load, alters ubiquitin conjugation, and can influence proteasome function. Recent integrative analyses emphasize that effective therapies must target multiple nodes restoring trophic signaling to restrain FOXO, dampening maladaptive NF-κB activation, and stabilizing calcium and mitochondrial homeostasis to prevent ROS-driven amplification of atrophy [8,14,76].

### 4.2. NF-κB and FOXO Signaling

Transcriptional control of muscle atrophy is largely governed by the NF-κB and FOXO families. NF-κB is rapidly activated by inflammatory cytokines, Toll-like receptor signals, and redox perturbations, driving expression of ubiquitin–proteasome components, inflammatory mediators, and anti-anabolic genes. Recent reviews highlight that NF-κB is a direct effector of fast-twitch fiber atrophy, and that its experimental inhibition preserves fiber size and contractile function across multiple models. NF-κB also intersects with redox biology, as ROS both activate and are amplified by NF-κB signaling, creating self-reinforcing loops between oxidative stress and inflammatory catabolism [77,78]. FOXO transcription factors link reduced trophic signaling, such as diminished IGF-1/PI3K–Akt activity after denervation or inactivity, to activation of proteolytic pathways. When Akt is suppressed, FOXO proteins enter the nucleus and induce atrogin-1, MuRF1, and autophagy-related genes that drive ubiquitin-dependent and lysosomal degradation. Recent work shows that FOXO activity is further shaped by redox-sensitive regulators such as AMPK and SIRT1 and by mitochondrial stress signals; AMPK–SIRT1–mediated changes in FOXO acetylation can shift its transcriptional output toward either adaptive autophagy or catabolic atrophy. These refined models explain why similar stressors can yield divergent FOXO responses and highlight AMPK/SIRT1/FOXO modulators as potential therapeutic targets [79,80].

### 4.3. Calcium Homeostasis and ROS-Mediated Signaling

Calcium homeostasis forms the key link between synaptic dysfunction, SR instability, and downstream proteolytic and mitochondrial injury. Oxidative modification of RyR1 enhances SR Ca^2+^ leak, elevating basal cytosolic Ca^2+^ and activating calpains, calcineurin, and other Ca^2+^-dependent pathways that drive protein degradation and transcriptional reprogramming. Excess Ca^2+^ uptake by mitochondria triggers permeability transition, membrane depolarization, and ROS bursts. Recent studies show that oxidative post-translational modifications of RyR1 destabilize its regulatory subunits and increase leak, while preventing RyR1 oxidation reduces Ca^2+^ dysregulation and protease activation in denervation and disease models. Thus, calcium imbalance transforms synaptic or activity deficits into a self-amplifying cascade of proteolysis and mitochondrial failure [8,81]. At a finer scale, cross-talk among these pathways exposes additional therapeutic targets. NF-κB and FOXO can co-regulate subsets of atrophy-related genes and microRNAs, while SIRT1-mediated FOXO deacetylation shifts its output between adaptive autophagy and maladaptive UPS activation. Mitochondrial quality control, including PINK1/Parkin-dependent mitophagy, OPA1/MFN-regulated fusion dynamics, and PGC-1α–driven biogenesis, is influenced by both redox and calcium signals, and its impairment elevates ROS production and increases proteolytic burden. Studies show that enhancing mitophagy or PGC-1α activity can redirect transcriptional programs toward resilience, indicating that mitochondrial-targeted interventions may exert broad benefits across these interconnected networks [8,78,82]. The neuromuscular junction is an active participant in this cross-talk. Synaptic instability reduces action potential delivery to muscle, diminishing calcium transients and ATP demand; this, in turn, impairs mitochondrial turnover and antioxidant capacity, fostering a redox environment that destabilizes synaptic proteins and the agrin–LRP4–MuSK axis. Oxidative modification of NMJ components thus both mirrors and accelerates the broader atrophy program. Translationally, therapies targeting the UPS, NF-κB/FOXO signaling, or calcium handling should be assessed not only for effects on muscle mass but also on synaptic integrity and motor unit performance, as highlighted in Table 3. Recent studies increasingly use integrated outcomes force generation, motor unit number, NMJ morphology, and mitochondrial function to capture these interdependencies [14,78,83].

## 5. Stem Cell-Based Therapeutic Strategies

Regenerating lost or atrophied skeletal muscle with cell-based therapies rests on a deceptively simple idea: replace or rejuvenate the cells that build and maintain muscle. In practice, however, success requires more than delivering a myogenic cell; it requires restoring a supporting niche, re-establishing vascular and neural connections, and controlling inflammation and redox stress during the vulnerable engraftment window. Over the last three years, the literature has made important strides: clearer mapping of satellite cell heterogeneity and niche signals, improved protocols to derive and mature myogenic progenitors from pluripotent sources, and new strategies that exploit the paracrine actions of MSCs and their extracellular vesicles. But the same body of work also underscores persistent barriers to long-term engraftment in intact muscle, inadequate reinnervation, immune and fibrotic responses, and manufacturing scale-up that must be solved if these therapies are to reach clinical impact [84,85,86].

### 5.1. Satellite Cells and Muscle Regeneration Capacity

Satellite cells, the resident MuSCs, are the first-line biological option for regenerative repair because they are intrinsically programmed to seed new myofibers and replenish the stem cell pool. Recent research has enhanced our understanding of MuSCs, transforming our perspective from a singular, uniform population to a diverse collective characterized by unique transcriptional states, regenerative capacities, and niche requirements. Single-cell transcriptomics and transplantation studies have revealed subsets that are primed for quiescence, rapid activation, self-renewal, or differentiation, and have clarified how aging and chronic disease skew this balance toward reduced self-renewal and senescence. Critically, techniques that preserve the native niche or recreate it during transplantation using supportive extracellular matrix components, endothelial co-transplants, or tailored hydrogels substantially improve MuSC engraftment and functional contribution to host muscle in recent preclinical reports. These studies make the point that successful MuSC therapy is as much about engineering an amenable microenvironment as it is about the cells themselves [87,88,89].

### 5.2. Mesenchymal Stem Cells and Paracrine Effects

MSCs are gaining interest primarily for their paracrine actions rather than direct myofiber fusion. They modulate immune activity, secrete trophic and angiogenic factors, and release extracellular vesicles (EVs) enriched in microRNAs and proteins that enhance MuSC function and mitochondrial resilience. Recent studies show that MSCs or their EVs improve muscle mass, reduce fibrosis, and mitigate denervation-induced degeneration in preclinical models. MSCs also offer practical advantages, straightforward isolation, low immunogenicity, and established GMP workflows, making them attractive for therapies focused on boosting endogenous repair rather than full tissue replacement. However, their benefits are often transient, as MSCs do not reliably replenish the MuSC pool, and durable outcomes likely require concurrent restoration of innervation and niche structure [90,91].

### 5.3. Induced Pluripotent Stem Cells (iPSCs) for Muscle Repair

Induced pluripotent stem cells (iPSCs) and iPSC-derived myogenic progenitors (iMPs) offer a scalable, patient-specific source of myogenic cells and have advanced quickly. Improved protocols using transient myogenic factor expression, small-molecule patterning, and refined purification now produce iMPs with strong fusion capacity and fetal-to-adult-like transcriptional profiles. Recent studies show that iPSC-derived progenitors can engraft and generate human myofibers in mice, with the host environment supporting further maturation. There is growing interest in engineering iMPs to deliver complementary functions—such as antioxidant enzymes or neurotrophic factors to enhance survival and integration. These developments position iPSC derivatives as promising tools for genetic correction and autologous therapy, while highlighting ongoing concerns around residual pluripotent cells, genomic stability, and long-term tumorigenic risk [85,86,92]. Biomaterials and tissue-engineering platforms have become essential adjuncts to cell therapy. Decellularized matrices, anisotropic hydrogels, and aligned scaffolds provide structural cues that guide myofiber orientation, support vascular ingrowth, and enhance satellite cell retention. Recent studies show that combining myogenic cells with ECM components or pro-angiogenic factors improves engraftment and boosts force recovery in volumetric muscle loss models. Scaffolds can also be engineered to shape the redox environment by releasing antioxidants or Nrf2 activators, protecting transplanted cells during the early, stress-prone period. These bioengineered niches simultaneously address mechanical alignment, vascularization, redox balance, and immune modulation, thereby improving the overall success of regenerative strategies [26,88].

### 5.4. Challenges: Engraftment, Immune Compatibility, Long-Term Function

Despite a strong mechanistic rationale, several recurring translational barriers limit clinical progress. Long-term engraftment and stable replenishment of the MuSC pool remain inconsistent, reflecting loss of niche cues during expansion, epigenetic drift, and inflammatory scarring in host tissue. Functional recovery also depends on reinnervation and vascularization; grafts that add mass without restoring motor unit connectivity produce weak, poorly coordinated muscle. Approaches integrating neurotrophic factors, electrical stimulation, or aligned nerve conduits improve synaptic formation in animal models, but scalability to large human injuries is unproven. Immune compatibility and safety present additional hurdles: allogeneic MSCs are moderately tolerated, whereas iPSC-derived products require stringent screening for residual pluripotent cells, genomic instability, and vector-related immune responses. Finally, the lack of standardized manufacturing and potency assays complicates comparison across studies and slows regulatory advancement. Recent reviews repeatedly highlight these challenges [84,93,94]. The field is increasingly moving toward combined or hybrid approaches to overcome these barriers. Co-transplanting MuSCs with endothelial progenitors enhances vascularization and supports niche restoration, while MSC-derived EVs delivered alongside myogenic cells reduce inflammation and improve survival. Engineered iMPs that co-express neurotrophic or antioxidant factors show greater persistence and promote reinnervation in preclinical models. Parallel efforts aim to activate endogenous repair in situ using small molecules, EVs, or gene therapeutics to rejuvenate resident MuSCs and remodel the niche without the need for transplanted cells, potentially avoiding manufacturing and immunogenicity hurdles. Together, these synergistic strategies mark a shift from simple cell replacement toward reconstructing the broader motor unit and its microenvironment [85,89,90]. Clinical translation is progressing but remains measured. Early trials using myoblasts, MSCs, or acellular scaffolds for volumetric muscle loss and select dystrophies demonstrate safety and modest functional benefits, but consistent improvements in strength and motor control have been challenging to achieve. Regulatory pathways require clear evidence of potency, safety, and mechanism of action, and recent guidance highlights the need for standardized assays that assess not only myogenic differentiation but also paracrine and immunomodulatory activity. Moving forward, robust outcome measures including motor unit number, NMJ structure, mitochondrial function, and objective force assessments will be essential to show that cellular therapies provide true functional restoration rather than simply increasing tissue bulk [26,94].

## 6. Gene-Based Therapeutic Strategies

### 6.1. Antioxidant Gene Delivery

Antioxidant gene delivery aims to shift the redox set point within muscle fibers and their neighboring motor terminals by supplying enzymatic scavengers or transcriptional activators of endogenous antioxidant programs. Targeting catalase or superoxide dismutase (SOD) to mitochondria has proven effective in multiple preclinical models: mitochondrial-targeted catalase expression reduces hydrogen peroxide burden, preserves mitochondrial function, and improves muscle strength in models of chronic injury and disuse, demonstrating that locally increasing H_2_O_2_ clearance can be disease-modifying rather than merely symptomatic. Recent experimental work using muscle-directed delivery of a mitochondria-targeted catalase (mCAT) showed improved neuromotor outcomes in mice with chronic kidney disease and limb injury, underscoring the translational potential of organelle-specific antioxidant gene therapy [95]. Rather than delivering a single enzyme, other strategies seek to activate the cell’s own antioxidant program by targeting Nrf2 signaling through gene transfer or small-molecule-resistant transcriptional modulators; preclinical studies and repurposing reports indicate that Nrf2 activation can protect against denervation-induced NMJ loss and reduce atrophy, making it an attractive, pathway-level gene therapy target [96]. The major challenges for antioxidant gene delivery are vector targeting (achieving durable, muscle-restricted expression without off-target hepatic expression), appropriate intracellular compartmentalization cytosolic versus mitochondrial localization, and avoiding over-suppression of physiologic redox signaling that is necessary for adaptation.

### 6.2. Gene Editing Approaches (CRISPR/Cas9, Base Editing)

Gene editing has advanced rapidly and now offers precise ways to correct monogenic causes of atrophy or to modify endogenous regulators of redox and proteostasis. CRISPR-Cas systems remain at the forefront for Duchenne muscular dystrophy (DMD) and other heritable myopathies, where exon excision or reframing can restore dystrophin expression; reviews published within the last year summarize optimized delivery constructs, improved guide design, and evolving safety data that make in vivo editing increasingly plausible [97,98]. Base editing and prime editing add precision by enabling single-base changes without generating double-strand breaks, and recent mini-reviews underscore their promise for rare monogenic disorders affecting muscle [99]. For non-monogenic atrophy, gene editing can also be used to knock down or modulate negative regulators of muscle mass, editing regulatory elements of myostatin or atrophy-promoting E3 ligases, although this approach is at an earlier preclinical stage and raises distinct safety and ethical considerations about durable, irreversible genomic edits in post-mitotic tissue.

### 6.3. AAV and Other Vector Systems for Muscle-Targeted Delivery

Adeno-associated virus (AAV) vectors remain the dominant delivery platform for muscle-directed gene therapies because of their tropism for skeletal muscle and proven clinical track record. The field has learned important lessons about dose, immunogenicity, and toxicity from the recent wave of neuromuscular gene therapies: clinical deployment of AAV micro-dystrophin for DMD established regulatory feasibility for systemic muscle gene transfer but also highlighted safety risks, including severe liver toxicity reported in post-approval cases, which demand stricter patient selection, immune management, and dosing strategies [100,101]. Contemporary reviews emphasize optimizing capsids for lower-dose efficacy, exploring tissue-restricted promoters, and combining transient immunomodulation with vector administration to reduce anti-AAV responses and hepatotoxicity [101,102]. RNA-based approaches, such as antisense oligonucleotides (ASOs), siRNA, and splice-modulating compounds, provide a less-permanent but clinically practical route to modulate gene expression. ASOs have matured clinically in DMD and SMA and are being used experimentally to downregulate atrophy-promoting targets or to alter splicing of genes that influence oxidative balance and muscle metabolism. The advantage is reversibility and dose control; the downside is frequent dosing and delivery hurdles in large muscles. Recent reviews summarize improved chemistries and systemic delivery methods that increase muscle uptake and reduce off-target effects, making RNA therapies an attractive complement or stopgap to durable gene transfer [103,104]. Safety, manufacturing, and durability are the limiting realities. AAV-mediated therapies face dose-dependent toxicities and pre-existing immunity; systemic high-dose exposure required to reach large muscle mass can provoke hepatic and immune adverse events, as documented in recent clinical program reviews and case reports [101]. Gene editing carries risks of off-target changes and long-term consequences of permanent edits, mandating rigorous preclinical safety pipelines and emerging in vivo toxicity assays. RNA-based therapeutics minimize permanence but demand optimized delivery platforms for durable benefit. On the manufacturing side, producing high-quality viral vectors and gene-modified cell products at clinical scale remains costly and technically challenging; recent reviews describe efforts to improve capsid engineering, manufacturing yields, and potency assays to meet regulatory expectations [101,102].

### 6.4. Modulation of Atrophy-Related Pathways (Myostatin, MuRF1, Atrogin-1)

Beyond replacing or editing genes, a promising class of gene-based interventions aims to modulate the regulatory networks that drive atrophy. Gene delivery of dominant-negative forms of NF-κB subunits, RNA interference against MuRF1 or atrogin-1, or AAV-mediated expression of IGF-1 isoforms have shown the capacity to blunt proteolysis and preserve muscle mass in animal models. These approaches demonstrate that altering signaling fluxes rather than correcting a structural protein can yield functional benefits. However, they require precise spatiotemporal control because the targeted pathways (NF-κB, FOXO, IGF1) play pleiotropic roles in immunity, metabolism, and growth; chronic suppression can therefore have adverse systemic effects. Recent preclinical reports illustrate both efficacy and cautionary signals, arguing for regulatable expression systems or cell-restricted delivery to minimize collateral toxicity [105,106]. A key translational insight from the literature was that combination gene approaches pairing antioxidant gene delivery with trophic factor expression, or combining editing of structural genes with co-delivery of Nrf2 activators, often outperform single-target therapies in preclinical models. Co-delivery can be achieved through dual-AAV strategies, bicistronic constructs, or by combining gene therapy with small molecules that prime the tissue, for instance, transient Nrf2 activation prior to cell- or gene-based engraftment. A concise summary of Stem cell and gene-based therapeutic strategies for skeletal muscle atrophy is provided in Table 4. Such synergy can address multiple failure modes simultaneously: improving redox resilience, supporting mitochondrial quality control, and maintaining synaptic integrity during the vulnerable engraftment or editing window [95].

## 7. Synergistic Approaches and Emerging Directions

### 7.1. Combining Stem Cell Therapy with Gene Modulation

**Figure 2:**
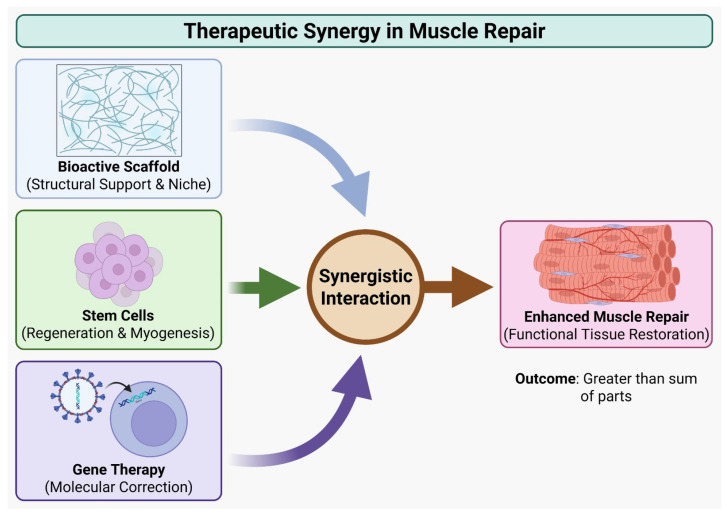
Translational pipeline and challenges.

Rebuilding a functional motor unit after atrophy requires integrated rather than single-modality approaches. As illustrated in Figure 2, the most effective strategies combine myogenic cells that rebuild fibers and provide trophic support, gene-based interventions that enhance host tissue resilience, scaffolds that restore architecture and guide reinnervation, and pharmacologic agents that control inflammation and oxidative stress during engraftment. The goal is to recreate a milieu in which new or rescued fibers can connect with vasculature and motor axons, maintain mitochondrial stability, and regain physiological activity. Recent preclinical and translational studies show that these components act synergistically and consistently outperform isolated interventions in restoring function [107,108]. Optimizing vector design and delivery is an important lever in combination strategies. Newly engineered AAV capsids with improved muscle tropism and reduced liver uptake enable effective host gene modulation at substantially lower doses, increasing safety when vectors are paired with cell grafts or immunomodulatory treatments. These advances allow targeted delivery of trophic or antioxidant genes to create a more supportive niche for transplanted cells while minimizing systemic immune complications. In parallel, scaffold-based delivery and localized electroporation offer non-viral alternatives that achieve strong regional expression without systemic AAV exposure [21,109].

### 7.2. Bioengineered Scaffolds and Exosome-Based Delivery Systems

Pairing myogenic cells, satellite cells, primary myoblasts, or iPSC-derived progenitors with biomaterial scaffolds is a well-established axis of synergy. Engineered anisotropic hydrogels and decellularized muscle matrices recreate the aligned architecture required for force transmission and provide pro-angiogenic cues that enhance engraftment, vascular infiltration, and contractile recovery in volumetric muscle loss models. When combined with progenitors, these scaffolds also support NMJ formation and improve early survival. Importantly, they can be engineered to modulate the local redox environment through slow-release Nrf2 activators or antioxidant chemistries shielding transplanted cells from oxidative bursts during the vulnerable post-implant period. These multimodal constructs thus simultaneously address mechanical, vascular, neural, and redox failure modes [27,88,110]. Extracellular vesicles (EVs) from MSCs and other supportive cells are emerging as a low-risk, off-the-shelf complement to cell and gene therapies. MSC-EVs deliver microRNAs, proteins, and metabolites that modulate inflammation, enhance mitochondrial quality control, and prime resident satellite cells for regeneration; when combined with scaffolds or progenitor transplants, they reduce fibrosis, limit oxidative injury, and improve functional recovery in preclinical atrophy and injury models. Because EVs carry fewer immunogenic or tumorigenic risks than living cells, they are well suited for early-stage combination strategies, such as EV-loaded hydrogels that provide sustained pro-regenerative signaling during graft integration and reinnervation. However, EV heterogeneity and the lack of standardized potency assays remain significant challenges for clinical translation [90,111]. Gene modulation enhances cell-based therapies through two complementary mechanisms. First, engineering transplanted cells to secrete neurotrophic, antioxidant, or angiogenic factors improves their survival, supports reinnervation, and boosts local mitochondrial function compared with unmodified cells. Second, targeted gene delivery to host tissue using muscle-tropic AAV capsids or tissue-restricted regulatory elements can precondition the niche by activating antioxidant pathways such as Nrf2, dampening maladaptive inflammation, or transiently increasing trophic support for incoming grafts. Recent animal studies show that combining cell transplantation with localized AAV or scaffold-based plasmid release yields larger, better-innervated grafts than either modality alone, underscoring the synergy between gene and cell therapies when precisely timed and targeted [21,109,112]. Bioengineering advances now enable precise spatial and temporal control over regenerative cues. Bioprinting and magnetically aligned hydrogels can generate organized fiber bundles and microvascular channels that support vascular and axonal ingrowth, while organoid and assembloid platforms provide refined preclinical testbeds for optimization. These technologies allow targeted placement of gene-releasing depots, cell reservoirs, and conductive elements, enabling staged therapies such as scaffolds that initially deliver Nrf2 activators to limit oxidative stress, then present neurotrophic signals to guide reinnervation, and finally provide aligned mechanical cues for contractile maturation. Early studies show that such spatiotemporally patterned designs produce better motor unit integration and functional recovery than uniform scaffolds [113,114].

### 7.3. Pharmacological Support for Cell/Gene Therapies

Pharmacological support and rehabilitative timing are essential for holding combination therapies together. Short-course immune modulation can control anti-AAV and graft-directed responses, while temporally targeted antioxidants or Nrf2 activators blunt early oxidative stress without disrupting adaptive redox signaling. Likewise, staged mechanical or electrical stimulation promotes NMJ maturation and activity-dependent stabilization of regenerated fibers. Transient Nrf2 activation before implantation improves mitochondrial stability, reduces early ROS-driven graft loss, and accelerates reinnervation in rodent models. Early but progressive rehabilitation—such as electrical pacing coordinated with graft maturation helps align regenerating fibers with incoming axons and enhances functional recovery. Translational efforts are shifting toward modular, combinatorial strategies in which each component can be optimized independently yet deployed as an integrated therapy. This requires robust potency assays for cells and EVs, well-characterized release kinetics and safety for scaffold-delivered molecules or vectors, and validated biomarkers such as motor unit number estimation, NMJ imaging, and mitochondrial function tests to capture functional outcomes. It also demands careful navigation of regulatory complexity: combination products span cell, gene, and device classifications, making early engagement with regulators and coordinated preclinical packages essential. Recent reviews emphasize these priorities and outline emerging frameworks for first-in-human studies of integrated muscle-regenerative approaches [27,115]. Cells supply structural and paracrine support; gene tools reprogram host tissue and enhance molecular resilience; scaffolds restore architecture, guide reinnervation, and localize therapeutic delivery; and pharmacology with staged rehabilitation shapes the immune and redox environment while promoting activity-dependent maturation. Moving forward, translation will rely on rational, staged combinations supported by improved capsids, localized gene delivery, standardized EV and cell products, and bioengineered niches that protect mitochondria and NMJs during engraftment. Early preclinical results are promising, but the key challenge is transforming these controlled successes into reproducible and safe clinical protocols [26,109,111].

## 8. Translational Challenges and Clinical Perspectives

### 8.1. Preclinical Models and Their Limitations

Preclinical-to-clinical pipeline for muscle regenerative therapies, highlighting model systems (rodents, large animals, iPSC-derived organoids), safety considerations (immune responses, toxicity, tumorigenicity), and regulatory checkpoints.

Figure 2 illustrates that translating advances in neuromuscular biology, redox control, and regenerative medicine into safe, effective therapies for human muscle atrophy remains a major engineering and clinical challenge. Laboratory successes that stabilize NMJs, reduce mitochondrial ROS, or increase fiber mass do not automatically produce durable functional recovery, because human muscle is large, highly vascularized, densely innervated, and embedded in a complex immune environment. Preclinical models have grown more sophisticated from rodent denervation and disuse models to large-animal studies and human iPSC-derived tissues and organoids, yet each has limitations. Rodents enable rapid mechanistic testing but differ markedly from humans in scale, motor unit structure, and immune responses; large animals better approximate human biomechanics and immunology but are expensive and slow. Human organoids and engineered muscle constructs offer species-matched platforms for assessing toxicity, engraftment, innervation, and vector performance, and are becoming practical tools for optimizing dosing and scaffold design. A pragmatic translational path therefore integrates small-animal genetics, large-animal scaling, and human tissue models, though doing so increases regulatory complexity, timelines, and cost [107,108].

### 8.2. Safety, Efficacy, and Ethical Considerations

Safety considerations remain paramount and vary by modality. Systemic AAV delivery has shown genuine therapeutic potential for muscle-targeted gene therapy, but recent clinical and regulatory events have underscored dose-dependent and unpredictable toxicities. Reports of severe liver injury and rare fatalities in older or non-ambulatory patients receiving high-dose AAV micro-dystrophin vectors prompted FDA investigations and company safety communications, highlighting the need for liver-sparing capsids, lower effective doses, and careful peri-dosing immune management. These events reinforce a central challenge: achieving meaningful transduction across the large mass of human muscle often requires vector doses that risk systemic organ toxicity and immune-mediated complications. Consequently, vigilant monitoring for transaminitis, cytokine and complement activation, and long-term oncogenic risk, although insertional mutagenesis is rare with AAV, must be integral to trial design [116]. Cell-based therapies carry distinct but equally important safety considerations. MSCs have a long clinical track record and generally favorable acute safety, making them attractive for paracrine-based approaches; however, product heterogeneity, donor variability, and inconsistent potency assays contribute to variable efficacy and complicate regulatory evaluation. Induced pluripotent stem cell (iPSC)–derived myogenic progenitors address supply and personalization needs but introduce new risks: residual pluripotent cells, genomic instability during prolonged culture, and potential tumorigenicity require stringent release testing and long-term monitoring, as reflected in recent cGMP preclinical programs. Additional practical challenges, such as scalability, cryopreservation effects on potency, and the lack of standardized predictive potency assays, remain key bottlenecks for clinical translation [117,118]. Efficacy endpoints must reflect true motor unit restoration rather than simply increased muscle bulk. Prior trials showing hypertrophy without meaningful functional improvement highlight the limitations of mass- or biomarker-only outcomes. For neuromuscular therapies, regulatory-grade efficacy should include objective measures of force and endurance, motor unit number estimation or electromyography, imaging of NMJ or nerve–muscle connectivity when feasible, and mitochondrial or oxidative biomarkers tied to the therapeutic mechanism. Studies combining modalities such as cells with scaffolds or gene therapy with electrical stimulation should prespecify composite endpoints that capture myofiber strength, coordination, and durable reinnervation, not just short-term volumetric gains. Recent translational reviews emphasize the need for standardized outcome batteries that align mechanisms, preclinical metrics, and clinical endpoints to avoid inconclusive results that impede development [119]. Immunogenicity remains a major barrier across modalities. For gene therapy, pre-existing anti-AAV antibodies, T-cell responses to capsids or transgene products, and complement activation can reduce efficacy or trigger serious adverse events. Mitigation strategies include capsid engineering to evade neutralizing antibodies, transient immunosuppression at dosing, plasmapheresis or IgG-degrading enzymes in sensitized individuals, and favoring localized over systemic delivery when feasible. In cell therapies, immune rejection of allogeneic grafts and inflammatory fibrosis at the implantation site hinder engraftment. Approaches under development, such as HLA matching, hypoimmunogenic engineering of iPSC-derived products, and co-delivery of MSCs or EVs to modulate inflammation, show early promise but increase manufacturing and regulatory complexity [120,121]. Manufacturing, potency testing, and regulatory strategy pose major practical hurdles that are often underestimated. Clinical-grade viral vectors for systemic muscle therapy require scalable, low-contaminant production methods and validated potency assays that predict in vivo transduction and functional benefit. Likewise, cell therapies demand stringent release criteria for identity, purity, potency, and safety—including sterility, genomic stability, and absence of residual vectors or pluripotent cells. Combination products, such as cells with scaffolds or genes with biomaterials, must satisfy overlapping drug, biologic, and device regulations, increasing preclinical data requirements and complicating early clinical trial design. Effective translation therefore depends on early regulatory engagement, comprehensive preclinical packages, including biodistribution and toxicology across species, and robust post-market surveillance planning [20,112]. Ethical and access challenges loom large for high-cost regenerative and gene therapies. Pricing and reimbursement models developed for rare, single-dose gene therapies will be strained by combination approaches that integrate scaffolds, cells, vectors, and prolonged rehabilitation. Issues of equitable access, long-term follow-up, and informed consent for durable genomic edits in non–life-threatening conditions must be addressed early in trial design and policy planning. Meaningful progress will require coordinated efforts among regulators, payors, and developers to create pathways that balance risk, benefit, and affordability [122].

### 8.3. Current and Upcoming Clinical Trials

Current and emerging clinical trials reflect both progress and caution. Studies of ECM scaffolds and biomaterials for volumetric muscle loss show good safety and occasional functional gains, but achieving consistent reinnervation and force recovery at scale remains challenging. MSC-based trials aimed at inflammatory modulation or local regenerative support are ongoing, and their variable efficacy highlights the need for standardized cell characterization and potency assays. Gene therapy programs for dystrophinopathies and other monogenic myopathies have achieved major regulatory milestones, yet high-dose systemic AAV–related safety events, including fatal liver toxicity in some trials, have led to stricter dosing and monitoring requirements, underscoring the narrow therapeutic window for whole-body gene delivery. In parallel, advanced preclinical and cGMP-ready iPSC-derived myogenic products are nearing first-in-human testing, emphasizing local delivery, rigorous release criteria, and staged immunomodulation to ensure safety. Overall, the clinical landscape suggests near-term potential in localized, scaffold-assisted reconstruction and cautiously executed systemic gene trials for select monogenic diseases, whereas broader applications to sarcopenia and disuse atrophy will likely require safer, lower-dose vectors, improved targeting, or non-viral local gene delivery [116,118,119,123,124].

## 9. Conclusions

Neuromuscular dysfunction and oxidative stress are interdependent drivers of skeletal muscle atrophy, where motor neuron integrity, NMJ stability, EC coupling, and mitochondrial redox balance together determine recovery versus proteolysis. Disturbance at any node RyR1 oxidation, axonal transport defects, mitochondrial Ca^2+^ overload rapidly activates UPS and FOXO/NF-κB pathways, committing fibers to atrophy unless the neural and metabolic milieu is restored. Exercise remains foundational but is often insufficient, making combinatorial strategies, cell and gene therapies, engineered scaffolds, EVs, and pharmacological modulators the most promising translational path. Progress requires human-relevant models, iPSC organoids, engineered constructs, and large animals, paired with biomarkers such as NMJ imaging, mitochondrial metrics, RyR1 oxidation, and redox signatures to guide development. Advances in vector design, delivery systems, and standardized cell/scaffold platforms are critical for safety and durability, while early clinical trials should prioritize localized applications, adaptive designs, and rigorous immune/toxicity monitoring. Near-term opportunities lie in scaffold-enhanced repair of volumetric muscle loss, MSC-EV adjuncts, and redox preconditioning, whereas systemic interventions for sarcopenia or myopathies will depend on safer vectors, scalable trophic delivery, and equitable access frameworks.

## Figures and Tables

**Table 1:** Neuromuscular mechanisms contributing to skeletal muscle atrophy.

Mechanism	Key Features	Pathological Consequences	Evidence Type	References
Motor neuron degeneration	Reduced axonal transport; loss of neurotrophic signaling (BDNF/TrkB, GDNF/RET); impaired synaptic vesicle cycling	Denervation, impaired excitation, rapid fiber atrophy	Genetic, pharmacologic, clinical	[36,52,53]
NMJ instability	AChR fragmentation; agrin–LRP4-MuSK pathway disruption; synaptic ECM disorganization (laminin α4/β2, collagen Q)	Transmission failure; progressive synaptic dismantling	Genetic, imaging, mechanistic	[54]
EC coupling failure	RyR1 Ca^2+^ leak; calstabin1 dissociation; T-tubule and DHPR remodeling; SERCA impairment	Reduced contractile force, mitochondrial Ca^2+^ overload, ROS amplification	Genetic, pharmacologic, structural biology	[55,56]
Integrated neural–oxidative–Ca^2+^ interaction (replaces “Cross-talk”)	Neural inactivity suppresses activity-dependent antioxidant programs (PGC-1α, NRF1); enhances RyR1-mediated Ca^2+^ leak; increases mitochondrial ROS/RNS; activates proteolytic signaling (calpains, caspases, NF-κB)	Feed-forward degeneration loop involving excitability loss, oxidative injury, and Ca^2+^ dysregulation	Genetic, biochemical, electrophysiologic	[57,58,59,60]

**Table 2:** Major sources of RONS in skeletal muscle.

Source	Mechanism of RONS Production	Conditions Amplifying Output	References
Mitochondria	ETC electron leak (Complexes I/III)	Aging, disuse, denervation	[11,61]
NADPH oxidases (NOX2/NOX4)	Enzymatic superoxide generation	Inactivity, insulin resistance	[9,63]
Nitric oxide synthase (nNOS/eNOS)	Uncoupling produces superoxide and peroxynitrite	Sarcopenia, metabolic disease	[64,65]
Inflammatory enzymes	Myeloperoxidase, xanthine oxidase	Chronic inflammation, cachexia	[62]

**Table 3:** Key molecular pathways driving muscle atrophy and their regulatory cues.

Pathway	Major Regulators	Effect on Muscle	Redox/Neuro Input	Ref.
Ubiquitin proteasome system (UPS)	Atrogin-1, MuRF1, 26S proteasome	Myofibrillar protein degradation	Activated by oxidative stress (ROS/RNS), FOXO activation during inactivity, and denervation-induced suppression of antioxidant programs. Neural inactivity → FOXO de-repression; ROS → proteasomal gene upregulation	[13,76]
Autophagy-lysosome	Beclin-1, LC3, p62	Organelle and protein turnover	Stimulated by ROS bursts, mitochondrial dysfunction, and Ca^2+^ overload following denervation. Redox imbalance promotes autophagosome formation; inactivity-linked mitochondrial stress amplifies flux	[14,78]
NF-κB	Inflammatory cytokines, ROS	Induces catabolic and inflammatory genes	Redox-sensitive transcription factor activated by oxidative stress and denervation-induced inflammation. Neural inactivity increases cytokine signaling; ROS enhances IKK/NF-κB activation	[77,78]
FOXO	PI3K-Akt inhibition, AMPK/SIRT1	Transcription of atrophy genes	Activated when neural activity is reduced and ROS increases. Oxidative stress promotes FOXO nuclear translocation; inactivity lowers Akt signaling, enabling atrophy gene induction	[79,80]
Ca^2+^ signaling	RyR1 leak, calcineurin, calpains	Proteolysis, mitochondrial stress	Denervation reduces excitation–contraction coupling → aberrant Ca^2+^ leak; ROS destabilizes RyR1, amplifying Ca^2+^ release. Ca^2+^ + ROS together activate calpains and mitochondrial stress	[7,81]

Pathway

Major Regulators

Effect on Muscle

Redox/Neuro Input

Ref

**Table 4:** Stem cell- and gene-based therapeutic strategies for skeletal muscle atrophy.

Strategy	Mechanism of Action	Advantages	Challenges/Limitations	References
Satellite cells (MuSCs)	Myofiber repair, pool replenishment	Native myogenic program	Poor long-term engraftment, niche loss	[87,88,89]
MSCs and EVs	Paracrine immunomodulation, trophic signaling	GMP-ready, lower immunogenicity	Transient effects, limited fusion	[90,91]
iPSC-derived progenitors	Scalable, patient-specific myogenic source	Genetic correction possible	Tumorigenicity, manufacturing hurdles	[85,86,92]
Antioxidant gene delivery	mCAT, SOD, Nrf2 activation	Restores redox balance	Vector targeting, safety concerns	[95,96]
Gene editing (CRISPR, base editing)	Correct structural or regulatory genes	Durable correction	Off-target risks, ethical issues	[97,98,99]
AAV/RNA-based therapies	Gene replacement, splicing modulation	Clinical precedent (DMD, SMA)	Immunogenicity, dose toxicity	[100,101,102,103,104]

## Data Availability

No new data were created or analyzed in this study.

## Abbreviations

The following abbreviations are used in this manuscript:

ALS	Amyotrophic lateral sclerosis
AAV	Adeno-associated virus
AChR	Acetylcholine receptor
Akt	Protein kinase B
AMPK	AMP-activated protein kinase
ASO	Antisense oligonucleotide
ATP	Adenosine triphosphate
Ca^2+^	Calcium ion
CRISPR	Clustered regularly interspaced short palindromic repeats
DMD	Duchenne muscular dystrophy
EC coupling	Excitation–contraction coupling
ECM	Extracellular matrix
EVs	Extracellular vesicles
FOXO	Forkhead box O transcription factor
GMP	Good manufacturing practice
HLA	Human leukocyte antigen
iMPs	Induced myogenic progenitors
iPSCs	Induced pluripotent stem cells
MSC	Mesenchymal stromal/stem cell
MuSCs	Muscle stem cells (satellite cells)
NF-κB	Nuclear factor kappa-light-chain-enhancer of activated B cells
NMJ	Neuromuscular junction
Nrf2	Nuclear factor erythroid 2-related factor 2
nNOS/eNOS	Neuronal/endothelial nitric oxide synthase
NOX	NADPH oxidase
PGC-1α	Peroxisome proliferator-activated receptor gamma coactivator 1-alpha
PTP	Permeability transition pore
RNA	Ribonucleic acid
RONS	Reactive oxygen and nitrogen species
RyR1	Ryanodine receptor type 1
SIRT1	Sirtuin 1
SMA	Spinal muscular atrophy
SOD	Superoxide dismutase
UPS	Ubiquitin–proteasome system
